# Bisphosphonate Use in Acute Spinal Cord Injury: A Focused Systematic Review on Zoledronic Acid

**DOI:** 10.7759/cureus.85755

**Published:** 2025-06-11

**Authors:** Sojeong Mun, Roopa Chalasani, Gilles Van de Vel, Pranav S Shukla, Shahab Ud Din Zia, Lubna Mohammed

**Affiliations:** 1 Physical Medicine and Rehabilitation, Hallym University College of Medicine, Chuncheon, KOR; 2 Research, Wake Forest Institute for Regenerative Medicine, Winston-Salem, USA; 3 Hospital Medicine, King's Mill Hospital, Mansfield, GBR; 4 Medicine, Grant Medical College and Sir JJ Group of Hospitals, Mumbai, IND; 5 Medicine and Surgery, Pak International Medical College, Peshawar, PAK; 6 Internal Medicine, Dr VRK Women's Medical College, Hyderabad, IND

**Keywords:** acute spinal cord injury, bisphosphonate, bone density, bone loss, zoledronic acid

## Abstract

Spinal cord injury (SCI) causes significant bone loss as a long-term complication, increasing fracture risk and healthcare costs. Bisphosphonates are widely studied for mitigating bone loss since they can prevent fractures and preserve rehabilitation potential in acute SCI patients. Zoledronic acid, in particular, stands out due to its high potency, dosing convenience, and better patient adherence. This review aims to evaluate the efficacy of bisphosphonates, particularly zoledronic acid, in mitigating bone loss in acute SCI. A systematic review was conducted following the Preferred Reporting Items for Systematic Reviews and Meta-Analyses (PRISMA) 2020 guidelines using PubMed, ScienceDirect, PubMed Central (PMC), Google Scholar, and Cochrane Central Register of Controlled Trials (CENTRAL) databases. Studies from January 1, 2016, to December 31, 2024, on bisphosphonate use within six months of SCI were included. Randomized controlled trials using zoledronic acid as the intervention and meta-analyses or systematic reviews covering all bisphosphonates were selected based on predefined inclusion and exclusion criteria. Eight studies, comprising three meta-analyses and five randomized controlled trials with 729 participants, were selected after quality assessment using Assessment of Multiple Systematic Reviews 2 (AMSTAR 2) and a revised Cochrane risk of bias tool for randomized trials (RoB 2). Seven studies demonstrated significant bone mineral density (BMD) improvements at the total hip, four at the lumbar spine, two at the trochanter, and five at the femoral neck. Two meta-analyses performed subgroup analyses of zoledronic acid, demonstrating substantial bone loss reduction, although comparisons with other bisphosphonates were lacking. Reductions in bone resorption markers, such as C-terminal telopeptide (CTX), were observed in five studies. While zoledronic acid exhibits strong anti-resorptive effects and allows for practical intravenous administration, its limited impact on bone formation markers, such as P1NP, and inconsistent BMD improvements across skeletal sites require further investigation. Future studies should assess long-term outcomes, utilize advanced imaging techniques, and directly compare zoledronic acid with other bisphosphonates.

## Introduction and background

Spinal cord injury (SCI) refers to damage to the spinal cord that results in temporary or permanent changes in motor, sensory, or autonomic function, posing a significant public health burden due to its high complication and disability rates. The incidence rate of SCI remains high, estimated at 23.77 per million people, while the demographic distribution of SCI patients continues to shift [1]. Acute SCI is predominantly caused by vehicular accidents, crush injuries, falls, diving, and medical complications [2].

Beyond the physical trauma, more than 20% of patients experience persistent psychological disorders, such as depression or anxiety, beyond six months post-discharge [3]. In patients with chronic SCI, probable major depressive disorder was found in 21% of patients at one year post-injury, with a slight decrease to 18% at five years post-injury [4]. In addition to psychological burdens, SCI imposes substantial financial challenges. The mean cost for acute care can reach up to 600,000 dollars, and the cost for inpatient rehabilitation can reach up to 400,000 dollars. The cumulative financial impact during the first year following injury can amount to 1.2 million dollars [5]. Due to its serious impact, it is crucial to understand SCI, both its management and potential complications, such as osteoporosis.

Comprehensive management of these patients requires not only emergency surgical intervention for the initial injury but also careful consideration of subsequent rehabilitation strategies and long-term quality-of-life outcomes [6]. Contemporary rehabilitation protocols for SCI encompass various therapeutic modalities, including range-of-motion exercises, strength training, bed mobility training, and transfer exercises, complemented by locomotor rehabilitation. Early and intensive mobilization is a fundamental principle in SCI rehabilitation protocols [7]. Along with these rehabilitation strategies, it is also essential to ensure long-term quality of life by managing secondary conditions effectively.

Osteoporosis is a severe and well-known long-term complication of SCI, characterized by distinctive patterns of bone loss. During the initial weeks post-injury, according to Thakkar et al.'s study on Indian patients, C-terminal telopeptide (CTX), a bone resorption biomarker, rises significantly above the normal range, peaks around the third month of injury with variable timing, and remains elevated beyond six months [8]. This accelerated bone loss occurs predominantly below the injury level, with studies indicating more pronounced bone loss in the knee compared to the hip during the first four to six months post-injury, although this pattern varies significantly among individuals [9]. The neurogenic bone loss associated with SCI extends beyond localized effects, leading to systemic disruptions in neural pathways, including 4-1BB signaling, receptor activator of nuclear factor kappa-B ligand (RANKL) signaling, and Wnt signaling, which affect bone remodeling. SCI also leads to compromised bone vascularity, which limits healing capacity, rehabilitation efficacy, and bone remodeling processes [10].

The underlying mechanism of bone loss after SCI involves dysregulation of bone homeostasis, primarily through osteoclast activity. Prolonged disuse and aberrant osteoclast function disrupt the equilibrium between bone formation and resorption, resulting in accelerated sub-lesional bone resorption and decreased bone density [11]. In the immediate post-SCI period, histomorphometric analyses of bone biopsies initially show a surge in both osteoblast and osteoclast activity. This pattern subsequently changes to increased osteoclastic activity and suppressed osteoblastic activity during the first several months after injury. As SCI progresses to its chronic phase, osteoblastic function returns to baseline levels, but elevated osteoclastic activity and bone resorption persist [12].

During the acute phase, bone loss can progress at 2-3% monthly and persist for three to seven years post-injury [13]. In contrast, chronic SCI presents a more gradual pattern of bone loss, potentially influenced by lifestyle factors such as vitamin D deficiency [14,15]. Bone loss in SCI patients is highlighted by an elevated risk of fragility fractures, particularly in the distal femur and proximal tibia, which leads to higher morbidity, mortality, and healthcare costs [11]. Given that osteoporosis imposes a substantial burden on SCI patients, it has been thoroughly studied to identify effective and practical therapeutic agents.

Bisphosphonates are widely employed for osteoporosis prevention through osteoclast inhibition. Among various analogs, traditional oral bisphosphonates require patients to maintain an upright position for at least 30 minutes post-administration, which is challenging for acute SCI patients who require supine positioning [16]. Conversely, intravenous zoledronic acid, a third-generation nitrogen-containing bisphosphonate, offers several advantages, including greater anti-osteoclastic potency compared to alendronate and pamidronic acid, along with the avoidance of gastrointestinal complications associated with oral administration [6]. Furthermore, because it is the only bisphosphonate administered once annually, it can significantly improve long-term patient compliance [9]. It is necessary to focus on specific analogs, particularly zoledronic acid, to develop effective treatment protocols and enhance outcomes in the management of SCI-related osteoporosis.

This systematic review synthesizes three meta-analyses and five randomized controlled trials to evaluate the efficacy of bisphosphonates, with a particular emphasis on zoledronic acid in managing acute SCI. While several systematic reviews and meta-analyses have investigated bisphosphonate use in acute SCI, these studies have typically covered various agents, including zoledronic acid, alendronate, and pamidronic acid [6,11,17]. This systematic review aims to address this limitation by focusing specifically on zoledronic acid interventions in randomized controlled trials. To provide a comprehensive perspective, this review also incorporates data from meta-analyses examining multiple bisphosphonates.

## Review

Methods

The efficacy of early bisphosphonate administration in acute SCI is the subject of this systematic review. This review adhered to the Preferred Reporting Items for Systematic Reviews and Meta-Analyses (PRISMA) 2020 guidelines [18]. In addition, this review did not conduct a new meta-analysis; instead, it provided a descriptive summary of the statistical findings reported in the included studies.

Database and Keywords

Through PubMed, ScienceDirect, PubMed Central (PMC), Google Scholar, and Cochrane Central Register of Controlled Trials (CENTRAL), keywords and medical subject heading (MeSH) terms were utilized to search relevant articles. MeSH terms such as "spinal cord injury", "acute spinal cord injury", "bone density conservation agents", "bisphosphonates", "diphosphonates", "bisphosphonate pharmacology", "bisphosphonate administration and dosage", "bisphosphonate therapeutic use", "bisphosphonate supply and distribution", "zoledronic acid", "alendronate", and "bone loss" were used. The Boolean method was utilized to refine search results by combining keywords and MeSH terms. Articles identified through this systematic process were assessed for relevance and eligibility. Duplicates or follow-up reports based on the same sample were manually removed during screening. Initially, titles and abstracts were evaluated to exclude irrelevant studies, followed by a thorough review of the full-text articles for further exclusion. A detailed description of the search strategy is demonstrated in Table 1.

**Table 1:** Details of the search strategy used PMC: PubMed Central; CENTRAL: Cochrane Central Register of Controlled Trials

Database	Search Strategy	Filters Applied	Results
PubMed	("Spinal Cord Injuries"[Mesh]) AND ("Bisphosphonates" OR ( "Diphosphonates/administration and dosage"[Mesh] OR "Diphosphonates/pharmacology"[Mesh] OR "Diphosphonates/standards"[Mesh] OR "Diphosphonates/supply and distribution"[Mesh] OR "Diphosphonates/therapeutic use"[Mesh] ) OR ( "Zoledronic Acid/administration and dosage"[Mesh] OR "Zoledronic Acid/pharmacology"[Mesh] OR "Zoledronic Acid/therapeutic use"[Mesh] ) OR ( "Alendronate/administration and dosage"[Mesh] OR "Alendronate/pharmacology"[Mesh] OR "Alendronate/supply and distribution"[Mesh] OR "Alendronate/therapeutic use"[Mesh] ) OR ( "Bone Density Conservation Agents/administration and dosage"[Mesh] OR "Bone Density Conservation Agents/pharmacology"[Mesh] OR "Bone Density Conservation Agents/standards"[Mesh] OR "Bone Density Conservation Agents/supply and distribution"[Mesh] OR "Bone Density Conservation Agents/therapeutic use"[Mesh] )) NOT ("Multiple myeloma" OR "Metast*")	2016–2024, Full text, English, Humans	35
ScienceDirect	Acute Spinal Cord Injury AND (Bisphosphonate OR Zoledronic acid OR Alendronate) AND (Bone loss OR Bone density) NOT (Multiple Myeloma OR Metastasis OR Animal)	2016–2024, Research articles, Medicine & Dentistry	29
PMC	“Acute Spinal Cord Injury” AND (Bisphosphonate OR Zoledronic acid OR Alendronate) AND (Acute OR Early) NOT (Multiple Myeloma OR Metastasis OR Cancer OR Duchenne OR Animal OR Denosumab)	2016–2024	18
Google Scholar	“Spinal Cord Injury” AND (bisphosphonate OR zoledronic acid OR alendronate) AND (Acute OR Early) AND "Efficacy" AND "Bone loss" -Animal -Metastasis -Myeloma -Denosumab	2016–2024	75
CENTRAL	(Bisphosphonate OR Zoledronate OR Zoledronic acid OR Alendronate OR Alendronate) AND Spinal Cord Injury AND Acute	Added to CENTRAL from 2016 to 2024	13

Inclusion Criteria

The inclusion criteria for the study are as follows: Studies published between January 1, 2016, and December 31, 2024, in English, were considered. Eligible studies had to be peer-reviewed, focus on human subjects, and provide full-text access, including access to paid articles. Articles needed to specifically address acute SCI patients who were within six months from injury or received early bisphosphonate administration and could include participants of all ages. Randomized controlled trials that exclusively used zoledronic acid as the intervention for acute SCI were included. Additionally, meta-analyses or systematic reviews encompassing data on zoledronic acid, alendronate, or pamidronate were included in the analysis. The study designs were limited to randomized controlled trials, cohort studies, systematic reviews, and meta-analyses, which provided robust methodologies and reliable data.

Exclusion Criteria

The exclusion criteria eliminated articles that were irrelevant to the topic, such as those addressing chronic SCI, conditions unrelated to SCI, or conditions introducing confounding factors (e.g., multiple myeloma, spinal metastasis, or Duchenne muscular dystrophy). Studies with overly broad topics that did not specifically focus on bisphosphonate administration or its timing and those addressing other medications (e.g., denosumab) were excluded. Animal studies, gray literature, and duplicated studies across databases were also excluded. Specific study designs, such as case reports, case series, narrative reviews, or editorials, were also excluded.

Results

Study Selection and Quality Assessment

**Figure 1:**
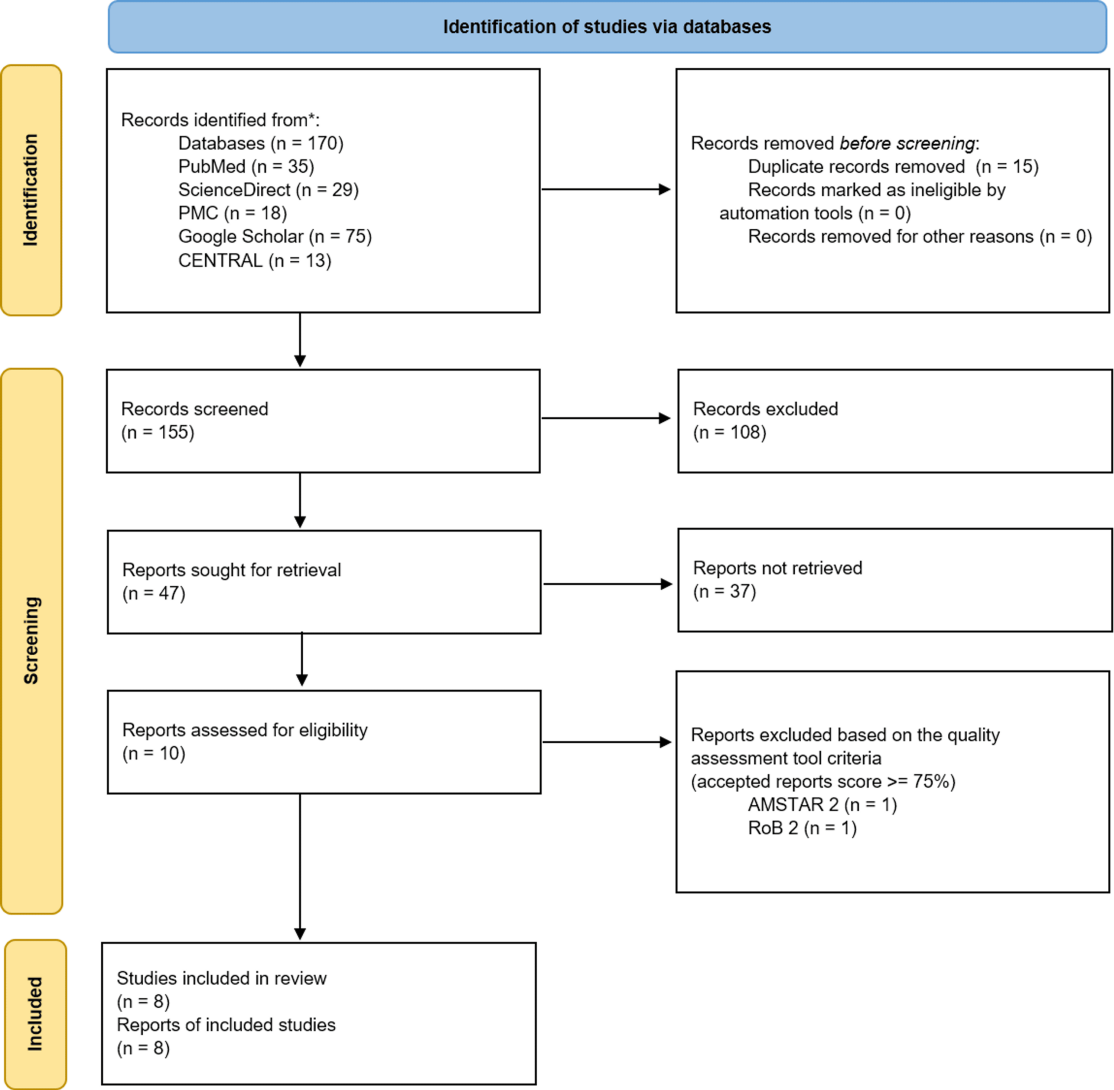
Flowchart demonstrating the selection process as per the PRISMA guidelines AMSTAR 2: Assessment of Multiple Systematic Reviews 2 [19]; RoB 2: a revised Cochrane risk of bias tool for randomized trials [20]; PRISMA: Preferred Reporting Items for Systematic Reviews and Meta-Analyses [18]

A total of 170 results were obtained from five databases: PubMed, ScienceDirect, PMC, Google Scholar, and CENTRAL. After aggregating these results, 15 duplicates were removed, leaving 155 unique records. Of these, 108 were eliminated due to irrelevant titles and abstracts. The remaining 47 reports were thoroughly reviewed as full-text articles, where 37 studies were excluded. Next, the remaining 10 studies were assessed using quality assessment tools specific to their study designs. Systematic reviews and meta-analyses were assessed by Assessment of Multiple Systematic Reviews 2 (AMSTAR 2) [19], and randomized controlled trials were assessed by a revised Cochrane risk of bias tool for randomized trials (RoB 2) [20]. Eight studies with a low risk of bias were selected for inclusion in this review, comprising three meta-analyses and five randomized controlled trials. A flowchart illustrating the identification and screening processes used to select the final articles is presented in Figure 1.

Tables 2, 3 below summarize the quality assessment of the included studies. The final data collection date was December 23, 2024. Publication bias was not tested in all three meta-analyses due to the small number of included studies and the small incidence of SCI [6,11,17].

**Table 2:** Quality assessment of included meta-analyses using AMSTAR 2 Y = Yes, PY = Partial Yes, N = No AMSTAR 2: Assessment of Multiple Systematic Reviews 2 [19]; RoB 2: a revised Cochrane risk of bias tool for randomized trials [20]; PICO: population, intervention, comparison, and outcomes

AMSTAR 2	Ma et al. (2022) [6]	Wu et al. (2021) [17]	Xinghua et al. (2020) [11]
PICO Components	Y	Y	Y
Protocol	PY	Y	PY
Study Design Explanation	Y	Y	Y
Comprehensive Search Strategy	Y	Y	Y
Duplicate Study Selection	Y	Y	Y
Duplicate Data Extraction	Y	Y	Y
Details of Excluded Studies	Y	Y	PY
Description of Included Studies	Y	Y	Y
Risk of Bias (RoB) Assessment	Y	Y	Y
Funding Sources of the Included Studies	N	N	N
(If Meta-Analysis) Statistical Combination Method	Y	Y	Y
(If Meta-Analysis) Potential Impact of RoB Assessment	Y	Y	Y
Impact of RoB to the Results	Y	Y	Y
Heterogeneity	Y	Y	Y
Publication Bias	N	N	N
Conflicts of Interest	Y	Y	Y

**Table 3:** Quality assessment of included randomized controlled trials using RoB 2 + = Low risk, ! = some concerns RoB 2: A revised Cochrane risk of bias tool for randomized trials [20]; D1-D5: Domain 1-Domain 5; Domain 1: Bias arising from the randomization process; Domain 2: Bias due to deviations from intended interventions; Domain 3: Bias due to missing outcome data; Domain 4: Bias in measurement of the outcome; Domain 5: Bias in selection of the reported result(s) [20]

RoB 2	Edwards et al. (2021) [9]	Oleson et al. (2020) [21]	Goenka et al. (2020) [16]	Goenka et al. (2018) [22]	Schnitzer et al. (2016) [23]
D1	+	+	+	+	!
D2	+	+	!	!	+
D3	+	+	+	+	!
D4	+	+	+	+	+
D5	+	+	+	+	+
Overall	+	+	!	!	!

Bone Mineral Density (BMD)

All eight included studies measured BMD using dual-energy X-ray absorptiometry (DXA) to assess the efficacy of bisphosphonates in acute SCI patients. The evaluated bones included the lumbar spine, total hip, trochanter, femoral neck, distal femur, proximal tibia, and forearm. Three meta-analyses and two randomized controlled trials measured BMD at the lumbar spine, with four of these studies reporting statistically significant percentage changes compared to the control group [6,9,11,17,23]. Similarly, all three meta-analyses and four randomized controlled trials that measured BMD at the total hip reported significant changes [6,9,11,17,21-23]. For the trochanter, one meta-analysis and one trial both showed statistically significant BMD changes [11,21]. The femoral neck was assessed in two meta-analyses and four trials, with significant percentage changes observed in five of the studies [6,9,11,21-23]. Although two meta-analyses and two trials measured BMD at the distal femur, only one trial reported a significant change at four months post-infusion [6,17,21,23]. The proximal tibia and forearm were each evaluated in a single trial, with only the forearm showing significant changes at six and 12 months of follow-up [16,21].

Among the eight included studies, only the trial by Edwards et al. used computed tomography (CT) in addition to DXA at the epiphysis of the femur and tibia, measuring BMD, trabecular bone mineral content, cortical bone mineral content, bone volume, cross-sectional area, and torsional strength index. While bisphosphonate treatment showed a significant effect at the distal femur and proximal tibia after 12 months of follow-up, no meaningful changes were observed after 24 months, except for the epiphyseal cortical bone mineral content in the tibia [9].

Bone Turnover Biomarkers

Three meta-analyses and three randomized controlled trials measured procollagen type I N-terminal propeptide (P1NP), a bone formation marker, after bisphosphonate administration [6,9,11,17,21,23]. Except for the trial by Edwards et al., where the group receiving two annual infusions of zoledronic acid (ZA-ZA) and the group receiving placebo in year one followed by zoledronic acid in year two (P-ZA) showed a statistically significant decrease in P1NP compared to the placebo-only group (P-P) and the group receiving zoledronic acid followed by placebo (ZA-P), none of the studies showed statistically meaningful changes in P1NP after bisphosphonate infusion [6,9,11,17,21,23].

Two meta-analyses and three trials measured CTX, a bone resorption marker, and all five studies reported statistically lower CTX levels following bisphosphonate administration, although the time points varied across the studies [6,9,17,21,23]. In particular, the meta-analysis by Ma et al. and the trial by Schnitzer et al. showed that CTX levels were significantly reduced only after six months, but not after 12 months, of follow-up [6,23]. However, in Ma et al., when a study with higher baseline CTX levels (Oleson et al. [21]) was excluded, CTX also showed a statistically significant decrease after 12 months. In contrast, in the trial by Oleson et al., CTX was significantly reduced after one and four months but not after 12 months, while the meta-analysis by Wu et al. reported a statistically significant reduction in CTX at 12 months [17,21]. In the randomized controlled trial by Edwards et al., participants who received two annual infusions of zoledronic acid (ZA-ZA) showed a meaningful reduction in CTX compared to the placebo-only group (P-P) and the group receiving zoledronic acid followed by placebo (ZA-P), but only after 24 months, not at six or 12 months of follow-up [9].

Other markers were also measured to compare the bisphosphonate and control groups. In the meta-analysis by Xinghua et al., calcium levels were measured in conjunction with P1NP and showed no significant changes; however, this finding may have been biased due to the small number of participants included [11]. Edwards et al. measured bone-specific alkaline phosphatase (BSAP), a bone formation marker, which showed a significant reduction in the group receiving two annual infusions of zoledronic acid (ZA-ZA) compared to the placebo-only group (P-P) and the group receiving zoledronic acid followed by placebo (ZA-P), but only after 24 months of follow-up [9].

Baseline characteristics are summarized in Tables 4, 5.

**Table 4:** Baseline characteristics of included meta-analyses SCI: spinal cord injury; BMD: bone mineral density; ZA: zoledronic acid; AA: alendronate; PA: pamidronic acid; RCT: randomized controlled trial

Reference	Year	Number of Studies Included	Included Study Design	Participants	Total Sample	Intervention	Control	Outcome
Ma et al. [6]	2022	7 (5 ZA, 1 AA, 1 PA)	RCT	Adult SCI <4–6 months with low bone mass	165	Bisphosphonate	Placebo, routine care	% change of BMD, bone turnover markers
Wu et al. [17]	2021	9 (6 ZA, 2 AA, 1 PA)	RCT	Adult SCI with low bone mass	206	Early bisphosphonate	Placebo, routine care, calcium	% change of BMD, bone turnover markers
Xinghua et al. [11]	2020	6 (4 ZA, 1 AA, 1 PA)	RCT	SCI with low bone mass	147	Early bisphosphonate	Placebo, routine care	% change of BMD, bone turnover markers

**Table 5:** Baseline characteristics of included randomized controlled trials Sex M/F data reflect the total sample for Goenka et al. (2020) [16], and subgroup-specific sex distribution was not separately reported. y: year; m: month; d: day; ZA: zoledronic acid; AA: alendronate; PA: pamidronic acid; IV: intravenous

Reference	Year	Study Location	Intervention	Control	Sample	Subgroup	Age (y): Treatment Group	Age (y): Control Group	Sex M/F	Days (d) to Intervention	Follow-up (m)
Edwards et al. [9]	2021	USA	ZA 5 mg IV (per 12 months)	Placebo	60	Total	37.3 (15.9)	38.2 (15.2)	48/12	65.7 (25.9)	24
Oleson et al. [21]	2020	USA	ZA 5 mg IV (Single)	Placebo	15	Total	35.9 (12.63)	30.8 (9.91)	10/5	21	12
Goenka et al. [16]	2020	India	ZA 5 mg IV (Single)	Routine care	60	Quadriplegics	38.89 (15.05)	36.3 (14.75)	50/7	30.06 (12.22)	12
Goenka et al. [16]	2020	India	ZA 5 mg IV (Single)	Routine care	60	Paraplegics	29.73 (8.02)	33.7 (8.24)	50/7	23.18 (11.34)	12
Goenka et al. [22]	2018	India	ZA 5 mg IV (Single)	Routine care	60	Total	35.41 (13.45)	35.57 (13.12)	49/8	27.5 (12.2)	12
Schnitzer et al. [23]	2016	USA	ZA 5 mg IV (Single)	Placebo	16	Total	44.3 (16.3)	34.1 (15.5)	15/1	69 (49)	24

The outcomes of the included studies are outlined in Tables 6-8. Tables 6, 7 describe the measured BMD and bone turnover markers, respectively, and Table 8 summarizes the conclusions of each study.

**Table 6:** Percentage change of BMD in the included studies * indicates p<0.05 in forest plots for meta-analyses and comparisons to placebo in randomized controlled trials. Within each column, the first number represents the mean difference or mean value, with the 95% confidence interval or standard deviation shown in parentheses. BMD: bone mineral density; DXA: dual-energy X-ray absorptiometry; m: month; T: treatment group; C: control group; P: placebo; ZA: zoledronic acid; NA: data not reported

Reference	Year	Follow-up	Comparison	Lumbar Spine	Total Hip	Trochanter	Femoral Neck	Distal Femur	Proximal Tibia	Forearm
Ma et al. [6]	2022	6 m	T/C	4.6 (2.7, 6.47)*	10.96 (8.72, 13.2)*	NA	8.69 (6.21, 11.16)*	5.4 (-3.88, 14.69)	NA	NA
Ma et al. [6]	2022	12 m	T/C	3.71 (0.07, 7.35)	15.70 (13.45, 17.95)*	NA	12.27 (7.89, 16.64)*	2.82 (-4.21, 9.85)	NA	NA
Wu et al. [17]	2021	12 m	T/C	4.67 (2.93, 6.41)*	12.97 (9.92, 16.02)*	NA	NA	2.57 (-3.35, 8.48)	NA	NA
Xinghua et al. [11]	2020	6 m	T/C	NA	11.68 (8.88, 14.48)*	13.41 (3.05, 23.77)*	15.03 (9.05, 21.02)*	NA	NA	NA
Xinghua et al. [11]	2020	12 m	T/C	3.59 (0.18, 6.99)	16.19 (13.80, 18.58)*	18.15 (9.99, 26.31)*	8.81 (1.09, 16.52)*	NA	NA	NA
Edwards et al. [9]	2021	24 m	P-P	-1.19 (6.33)	-18.8 (7.19)	NA	-16.6 (6.64)	NA	NA	NA
Edwards et al. [9]	2021	24 m	P-ZA	3.23 (5.43)	12.0 (7.07)	NA	-12.4 (7.28)	NA	NA	NA
Edwards et al. [9]	2021	24 m	ZA-P	6.13 (3.23)*	8.11 (7.35)*	NA	-5.90 (6.96)*	NA	NA	NA
Edwards et al. [9]	2021	24 m	ZA-ZA	5.50 (2.83)*	4.31 (5.83)*	NA	-2.88 (4.13)*	NA	NA	NA
Oleson et al. [21]	2020	4 m	T/C	NA	0.92 (7.14)*	1.19 (7.77)*	0.97 (6.12)*	-0.78 (4.46)*	-0.24 (11.7)	NA
Oleson et al. [21]	2020	12 m	T/C	NA	-8.20 (3.64)*	-8.62 (4.70)*	-3.93 (5.60)*	-8.07 (5.2)	-4.54 (9.89)	NA
Goenka et al. [16]	2020	3 m	T/C	NA	NA	NA	NA	NA	NA	-0.002 (-0.009, 0.006)
Goenka et al. [16]	2020	6 m	T/C	NA	NA	NA	NA	NA	NA	-0.049 (-0.062, -0.036)*
Goenka et al. [16]	2020	12 m	T/C	NA	NA	NA	NA	NA	NA	-0.115 (-0.132, -0.097)*
Goenka et al. [22]	2018	3 m	T/C	NA	-0.05 (-0.07, -0.03)*	NA	-0.01 (-0.05, 0.03)	NA	NA	NA
Goenka et al. [22]	2018	6 m	T/C	NA	-0.12 (-0.15, -0.08)*	NA	-0.08 (-0.12, -0.03)*	NA	NA	NA
Goenka et al. [22]	2018	12 m	T/C	NA	-0.16 (-0.19, -0.12)*	NA	-0.13 (-0.18, -0.09)*	NA	NA	NA
Schnitzer et al. [23]	2016	6 m	T/C	2.4 (1.8)*	-3.52 (0.66)*	NA	-1.1 (3.5)	-3.3 (2.9)	NA	NA

**Table 7:** Serum bone turnover markers in the included studies * indicates p<0.05 in forest plots for meta-analyses and comparisons to placebo in randomized controlled trials. Within each column, the first number represents the mean difference or mean value, with the 95% confidence interval or standard deviation shown in parentheses. m: month; P1NP: procollagen type I N-terminal propeptide; CTX: C-terminal telopeptide; T: treatment group; C: control group; P: placebo; ZA: zoledronic acid; NA: data not reported

Reference	Year	Follow-up	Comparison	P1NP				CTX			
Ma et al. [6]	2022	6 m	T/C	-23.30 (-69.20, 22.60)				-0.36 (-0.48, -0.24)*			
Ma et al. [6]	2022	12 m	T/C	-13.99 (-56.99, 29.01)				-0.20 (-0.45, 0.05)			
Wu et al. [17]	2021	12 m	T/C	-21.01 (-45.44, 3.42)				-0.41 (-0.54, -0.28)*			
Xinghua et al. [11]	2020	12 m	T/C	-19.93 (-48.92, 9.06)				NA			
Edwards et al. [9]	2021	6 m	P-P	92.10 (42.00)				0.29 (0.14)			
Edwards et al. [9]	2021	6 m	P-ZA	104.00 (73.90)				0.34 (0.24)			
Edwards et al. [9]	2021	6 m	ZA-P	84.20 (75.50)				0.21 (0.11)			
Edwards et al. [9]	2021	6 m	ZA-ZA	70.80 (50.70)				0.15 (0.11)			
Edwards et al. [9]	2021	12 m	P-P	75.00 (30.60)				0.26 (0.14)			
Edwards et al. [9]	2021	12 m	P-ZA	90.10 (77.20)				0.36 (0.25)			
Edwards et al. [9]	2021	12 m	ZA-P	59.70 (31.90)				0.26 (0.14)			
Edwards et al. [9]	2021	12 m	ZA-ZA	55.60 (32.70)				0.15 (0.12)			
Edwards et al. [9]	2021	24 m	P-P	62.80 (26.30)				0.19 (0.11)			
Edwards et al. [9]	2021	24 m	P-ZA	49.80 (44.95)*				0.18 (0.19)			
Edwards et al. [9]	2021	24 m	ZA-P	74.20 (43.00)				0.22 (0.14)			
Edwards et al. [9]	2021	24 m	ZA-ZA	34.10 (16.60)*				0.10 (0.08)*			
Oleson et al. [21]	2020	1 m	T/C	99.40 (41.48)				0.52 (0.16)*			
Oleson et al. [21]	2020	4 m	T/C	140.40 (157.14)				1.08 (0.78)*			
Oleson et al. [21]	2020	12 m	T/C	NA				1.36 (1.25)			
Goenka et al. [16]	2020	-	NA	NA				NA			
Goenka et al. [22]	2018	-	NA	NA				NA			
Schnitzer et al. [23]	2016	6 m	T/C	67.3 (13.5)				0.36 (0.14)*			
Schnitzer et al. [23]	2016	12 m	T/C	50 (20.4)				0.19 (0.11)			

**Table 8:** Conclusions of the included studies SCI: spinal cord injury; P1NP: procollagen type I N-terminal propeptide; CTX: C-terminal telopeptide

Reference	Year	Conclusions
Ma et al. [6]	2022	Bone loss was mitigated by early bisphosphonate administration in the total hip, lumbar spine, and femoral neck of SCI patients, while there was no notable impact on the knee joint. No statistically significant difference in P1NP was present, while bisphosphonates showed benefit in reducing CTX. Further studies regarding the administration route of bisphosphonate should be considered.
Wu et al. [17]	2021	Bone loss was mitigated by early bisphosphonate administration in the total hip and lumbar spine, while there was no notable impact on the distal femur. No statistically significant difference in P1NP was present, while bisphosphonates showed benefit in reducing CTX. Early administration of bisphosphonate is safe. Further studies with larger samples and longer follow-ups should be considered.
Xinghua et al. [11]	2020	Bone loss was mitigated by early bisphosphonate administration in the total hip and lumbar spine. No statistically significant difference in P1NP was present. Early administration of bisphosphonate is safe.
Edwards et al. [9]	2021	After 12 months of follow-up, bone loss was mitigated by a single infusion of zoledronic acid in the total hip, femoral neck, distal femur, and distal tibia. However, only the total hip showed a significant benefit from the subsequent second infusion after 24 months compared to a single infusion. Both P1NP and CTX were decreased after 24 months of follow-up, while there were no significant differences after 12 months. Intravenous zoledronic acid is well tolerated and effective for acute SCI patients.
Oleson et al. [21]	2020	After 4 months of follow-up, bone loss was mitigated by a single infusion of zoledronic acid in the hip (total hip, trochanter, and femoral neck) and knee (distal femur). However, zoledronic acid showed benefits for the hip after 12 months. No statistically significant difference in P1NP was present, while bisphosphonates showed benefit in reducing CTX after 2 weeks and 4 months of follow-up.
Goenka et al. [16]	2020	After 12 months of follow-up, bone loss was mitigated by a single infusion of zoledronic acid in the forearm. Further studies with larger samples and longer follow-ups should be considered.
Goenka et al. [22]	2018	After 12 months of follow-up, bone loss was mitigated by a single infusion of zoledronic acid in the total hip and femoral neck. Further studies with larger samples and longer follow-ups should be considered.
Schnitzer et al. [23]	2016	After 6 months of follow-up, compared with the placebo, bone loss was mitigated by a single infusion of zoledronic acid in the total hip. In the lumbar spine, bone density increased significantly in the zoledronic acid group, while the placebo group showed a significant decrease. No statistically significant difference in P1NP was present, while bisphosphonates showed benefit in reducing CTX after 6 months of follow-up.

Discussion

Bone loss represents a significant sequela following acute SCI, characterized by a rapid decline in bone density and a higher risk of fractures below the neurological level of injury. Due to this, the use of bisphosphonate therapy to prevent and manage accelerated bone loss has become a crucial topic in clinical research. This discussion examines the role of bisphosphonates, with particular emphasis on intravenous zoledronic acid, in influencing BMD and bone turnover markers in acute SCI patients, synthesizing findings from the included studies.

Mechanisms, Administration Routes, and Adverse Events of Bisphosphonates: Focus on Zoledronic Acid

Bisphosphonates effectively inhibit osteoclast-mediated bone resorption and have evolved with continuous improvements in potency across generations. Following the development of the first-generation bisphosphonates, etidronic acid and clodronic acid, pamidronic acid and alendronate were introduced as the first nitrogen-containing bisphosphonates. Zoledronic acid and risedronate are the latest generation, featuring heterocyclic side chains [24]. In particular, zoledronic acid is a third-generation nitrogen-containing bisphosphonate featuring a heterocyclic imidazole ring, enhancing its binding affinity to hydroxyapatite and increasing its potency as an inhibitor of osteoclast activity [25].

**Figure 2:**
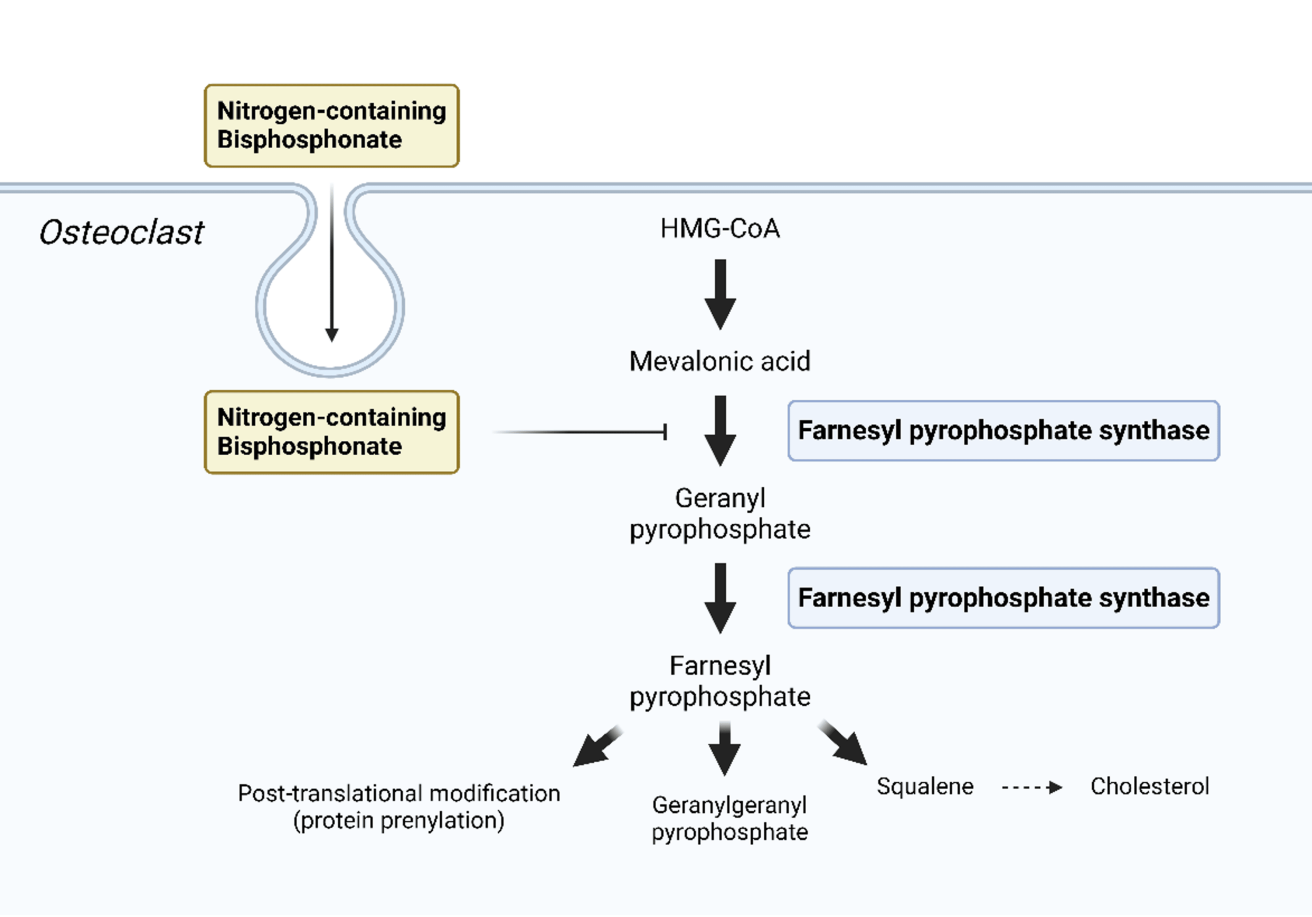
Mechanism of osteoclast inhibition by nitrogen-containing bisphosphonates Image created by the first author using biorender [27].

After selective endocytosis by osteoclasts, zoledronic acid binds to farnesyl pyrophosphate synthase (FPPS) within the mevalonic acid pathway. This inhibition prevents protein prenylation, which is required for small GTPase signaling proteins essential for cytoskeletal organization and vesicular trafficking in osteoclasts. Consequently, this disruption leads to loss of osteoclast function and induces apoptosis [26]. Figure 2 illustrates the mechanism by which nitrogen-containing bisphosphonates inhibit osteoclast activity.

Bisphosphonates are available in various administration routes and dosages. Nitrogen-containing bisphosphonates, such as alendronate and risedronate, are administered orally (alendronate 70 mg and risedronate 35 mg weekly or risedronate and ibandronic acid 150 mg each monthly). Recently, intravenous bisphosphonates, such as ibandronic acid (3 mg every three months) and zoledronic acid (5 mg annually), have been developed [28]. Studies have shown that annual intravenous zoledronic acid improves treatment adherence compared to other bisphosphonates, as patients prefer less frequent dosing [29]. Additionally, to prevent esophagitis, a common side effect of bisphosphonates, it is recommended to remain upright for at least 30 minutes (60 minutes for oral ibandronic acid specifically) after taking oral bisphosphonates [30,31]. As a result, adherence to oral bisphosphonates remains poor even with less frequent dosing. This potentially increases fracture risk by limiting bone density improvements and poses particular challenges for patients with acute SCI, who are often bedridden [31,32]. Consequently, intravenous bisphosphonates are generally preferred by patients with gastrointestinal intolerance or difficulty adhering to oral regimens [31]. Moreover, they are especially more practical for use in bedridden SCI patients because they bypass the challenges of oral administration.

The most common short-term side effects of bisphosphonates include gastrointestinal intolerance (particularly esophagitis), acute phase reaction, and uveitis. Esophagitis is caused by prolonged contact of the bisphosphonate tablet with the esophageal mucosa, which causes local irritation [28]. Acute phase response (APR) is more frequent in intravenous formulations and is defined as a flu-like reaction, including fever, chills, headache, myalgia, fatigue, and arthralgia, beginning within three days of infusion [9]. Ocular inflammation, such as uveitis, conjunctivitis, and scleritis, has rarely been reported in both oral and intravenous bisphosphonates [33]. Osteonecrosis of the jaw, atrial fibrillation, and bone turnover suppression are well-known long-term side effects of bisphosphonates [28]. Nevertheless, the long-term adverse events could not be identified in this systematic review, as we focused on bisphosphonate administration in the acute phase of SCI, including studies with follow-up periods of 24 months or less.

Among the included studies, two meta-analyses and two randomized controlled trials reported that bisphosphonates were well tolerated, with no notable adverse events [6,16,17,22]. In the meta-analysis by Wu et al., although a statistically significant difference in the incidence of fever was observed when analyzed separately, no significant difference in the overall occurrence of side effects was found between the bisphosphonate and control groups after pooling the studies [17].

Three trials included in this study have described adverse effects following zoledronic acid infusion [9,21,23]. In the randomized controlled trial by Edwards et al., with 24 months of follow-up, 411 adverse events were reported, including urinary tract infections, APR, and upper respiratory infections, with 53 classified as severe. However, none of the serious events were exclusively attributed to zoledronic acid, and the most common events were related to the underlying SCI itself. The incidence of APR was significantly higher compared to previous reports for postmenopausal women and men treated with zoledronic acid, although all cases were self-limited [9]. The other two trials reported fever, with a statistically higher incidence noted in the trial by Oleson et al. [21,23]. APR was the most commonly discussed side effect across studies reporting adverse events. Further research on APRs in acute SCI patients following zoledronic acid infusion is suggested.

Bone Mineral Density

BMD is currently the primary tool for diagnosing osteoporosis and predicting fracture risks, although it does not identify individuals who will experience a fracture [34]. The most widely used standard method to test BMD for osteoporosis diagnosis is DXA, which evaluates the mineral content of a patient's bone. T-scores, which determine whether a patient is osteopenic, are calculated by comparing the patient's BMD to the mean BMD of healthy young adults of the same gender and ethnic group and then dividing the difference by the standard deviation of the young adult population [35]. Table 9 provides the World Health Organization (WHO) definitions of osteoporosis and osteopenia in postmenopausal white women based on T-scores [36].

**Table 9:** WHO definition of osteoporosis and osteopenia by DXA Definitions of osteoporosis and osteopenia in postmenopausal white women based on T-scores by DXA [36]. Permission was obtained from the original publisher. WHO: World Health Organization; DXA: dual-energy X-ray absorptiometry

Diagnosis	T-score
Normal	T ≥ -1.0
Osteopenia	-2.5 < T
Osteoporosis	T ≤ -2.5
Established osteoporosis	T ≤ -2.5 with one or more fragility fractures

Recently, using Hounsfield units (HUs) from CT scans has been suggested as an alternative method to measure BMD and assess fracture risks without additional costs. HU measurement from CT scans has been revealed to correlate with BMD measured by DXA [37,38]. Studies have also described that it is possible to identify fracture risk in SCI patients, specifically in the femur and tibia, by measuring bone mineral content and volumetric BMD of integral, trabecular, and cortical bone [39,40].

This systematic review analyzed eight studies, examining the percentage changes in BMD measured by DXA after bisphosphonate administration at the following sites: lumbar spine, total hip, trochanter, femoral neck, distal femur, proximal tibia, and forearm. Statistically significant improvements in BMD were observed in most studies, particularly at the lumbar spine, total hip, trochanter, and femoral neck. Notably, the total hip consistently showed significant changes across all studies that measured it, underlining its potential as a reliable site to assess the efficacy of bisphosphonate [6,9,11,17,21-23]. In contrast, results for the distal femur and proximal tibia were limited. The forearm also showed meaningful changes, although it was relatively studied less frequently than other sites [6,16,17,21,23].

Conversely, in the randomized controlled trial by Edwards et al., significant effects were observed at the trabecular and cortical compartments of the femur and tibia when CT scans were used in addition to DXA after 12 months of follow-up. This trial was also the longest and the only study among the eight included studies tracking participants annually for 24 months, although the effect of bisphosphonates on both the femur and tibia was limited at the 24-month follow-up [9]. These gaps may occur due to the differences in the measurement tools and the amount of data available for each bone. Future studies are encouraged to include assessments of various bones using more imaging modalities, larger sample sizes, and long-term follow-ups to address these gaps.

Two of the three included meta-analyses conducted subgroup analyses on the potency of zoledronic acid; however, comparisons with other bisphosphonates, such as alendronate and pamidronic acid, were not specified [6,17]. According to Ma et al., zoledronic acid demonstrated significant differences in BMD at the lumbar spine, total hip, and femoral neck six months after administration, while significant differences were observed at the total hip and femoral neck after 12 months [6]. Similarly, in the meta-analysis by Wu et al., zoledronic acid showed significant changes in BMD in the lumbar spine and total hip [17]. Considering that all five randomized controlled trials that used zoledronic acid as an intervention reported significant changes in BMD, further studies with sufficient data to directly compare the potency of specific bisphosphonates are recommended.

Bone Turnover Biomarkers

While osteoclast inhibition by bisphosphonates is not directly measurable, their efficacy can be reliably assessed using biochemical markers in serum and urine [26]. The most commonly measured bone formation markers are P1NP, BSAP, and osteocalcin, while bone resorption markers include serum CTX, urinary N-terminal collagen telopeptide, and deoxypyridinoline [28]. P1NP is a marker of type 1 collagen secretion by osteoblasts, and BSAP is one of the products of four *ALP* genes, indicating bone anabolic activity. Osteocalcin, the most abundant non-collagen protein in bone, is also secreted by osteoblasts [41]. The most abundant protein component of the bone, type 1 collagen, produces C- and N-terminal telopeptides as its fragments. Deoxypyridinoline is a form of cross-link between hydroxylysine residues within collagens, predominantly found in the bone [41].

It has been reported that bisphosphonates decrease both bone formation and resorption markers, with zoledronic acid showing greater reductions in bone turnover markers compared to alendronate and risedronate [31]. Across the included studies, P1NP generally did not show statistically meaningful changes following bisphosphonate administration [6,9,11,17,21,23]. However, Edwards et al. reported a significant decrease in P1NP in groups that received two annual infusions of zoledronic acid (ZA-ZA) or placebo, followed by zoledronic acid (P-ZA), compared to the placebo-only group (P-P) and the zoledronic acid followed by placebo group (ZA-P). In the same study, BSAP was also statistically reduced after two annual infusions of zoledronic acid [9]. Furthermore, Delmas et al. concluded that the third infusion of zoledronic acid significantly increased the persistence of CTX reduction by 60%, without an associated increase in fracture risk, despite the reduction in P1NP [42]. This suggests that sequential infusions of zoledronic acid may have more effect on bone formation suppression. Future studies with follow-ups exceeding 24 months are encouraged to better assess the long-term impact of bisphosphonates, particularly zoledronic acid, and their persistence. The relationship between fracture risk and the reduction in bone formation markers induced by bisphosphonates should also be evaluated.

CTX was consistently decreased among the five studies that measured it [6,9,17,21,23], confirming the efficacy of bisphosphonates in reducing bone resorption in acute SCI patients. Nevertheless, the timing of CTX reductions varied. This might be due to the baseline heterogeneity and the inclusion of different types of bisphosphonates in the meta-analyses. The need for more extended follow-up studies is evident here as well, aligning with findings related to P1NP.

Unlike BMD, none of the three meta-analyses performed subgroup analyses specifically for zoledronic acid regarding bone turnover markers. Future studies should include comparisons with other bisphosphonate analogs to understand their differences in effects on bone turnover markers better.

Strengths

This systematic review included randomized controlled trials, cohort studies, systematic reviews, and meta-analyses, ensuring the inclusion of robust, high-level methodologies. By incorporating meta-analyses that examined various bisphosphonate analogs, along with subgroup analyses of zoledronic acid, as well as randomized controlled trials that exclusively used zoledronic acid as an intervention, this review highlights the efficacy of zoledronic acid for SCI-related osteoporosis, while comprehensively addressing the effects of other bisphosphonates and filling gaps from recent studies. Additionally, most studies globally assessed BMD and bone turnover markers to evaluate bone loss, enhancing the reliability of the findings and the correlation between bisphosphonate usage and osteoporosis following acute SCI. According to the PRISMA 2020 guidelines, the quality of all included studies was thoroughly assessed using established tools, such as AMSTAR 2 and RoB 2, with all studies receiving high-quality appraisals. Lastly, this review covers the adverse events of bisphosphonates in acute SCI patients, not only their efficacy, as an effort to maintain a neutral perspective for establishing practical treatment protocols.

Limitations

This systematic review has several limitations. First, the included studies were limited to those published between 2016 and 2024, which may have resulted in insufficient data due to the exclusion of foundational earlier studies. Since non-English studies were also excluded, the global applicability of this review may be limited. Additionally, we could not evaluate publication bias due to the small number of included studies. In fact, none of the meta-analyses assessed publication bias, likely because they also included a limited number of studies. Although not thoroughly assessed, publication bias may be increased, as this review excluded studies that specifically focused on the adverse effects of bisphosphonates in acute SCI patients, whereas we have discussed the adverse effects of the included studies. This can limit the reliability of the results and broad perspectives, and future studies are encouraged to include more studies with larger and more diverse participants.

Baseline characteristics, such as age, sex, race, and comorbidities, may have influenced the outcomes, but these factors were not consistently discussed across studies, which may limit the generalizability of the findings to a wider SCI population. Furthermore, the included studies varied in follow-up durations, timing of bisphosphonate administration after injury, dosages, number of administrations, bisphosphonate types, and imaging modalities such as DXA and CT, which may have influenced the comparability of results and made it challenging to isolate the effects of zoledronic acid. Future research should focus exclusively on zoledronic acid to provide more targeted insights, maintaining low heterogeneity by standardizing population characteristics, imaging modalities, and study designs.

This review could not assess the longer-term effects on fracture risk or delayed side effects due to the relatively short follow-up durations in the included studies. Although this review emphasized the importance of rehabilitation, it did not specifically evaluate functional or quality-of-life outcomes, which are critical for optimal rehabilitation of SCI patients. Confounding factors such as nutritional status, vitamin D and calcium levels, physical rehabilitation status, and American Spinal Injury Association (ASIA) levels were not considered despite their potential influence on BMD and bone turnover markers.

## Conclusions

This systematic review demonstrates that early administration of bisphosphonates, particularly zoledronic acid, mitigates bone loss in acute SCI effectively, although its efficacy depends on the site, patient characteristics, and the regimen used. Across the eight included studies, significant improvements in BMD were observed at the lumbar spine, total hip, trochanter, and femoral neck, with the most consistent results seen at the total hip. While the timing of reduction varied, all studies that assessed the bone resorption marker CTX reported significant decreases. Zoledronic acid emerges as a promising option for immobilized SCI patients among bisphosphonate analogs due to its strongest potency, intravenous administration, and less frequent dosing compared to oral alternatives. Future studies should focus on the long-term effects of bisphosphonates using larger samples and advanced imaging modalities while investigating the relationship between biochemical marker changes and fracture risk. Studies that thoroughly compare the efficacy and side effects of different bisphosphonate analogs are also recommended. Suggesting zoledronic acid as a practical treatment option for SCI-related bone loss, this review contributes to enriching the approach for the integration of pharmacological and rehabilitative care after acute SCI.
